# Non-*aureus* staphylococci and mammaliicocci isolated from bovine milk in Italian dairy farms: a retrospective investigation

**DOI:** 10.1007/s11259-023-10187-x

**Published:** 2023-08-10

**Authors:** Maria Filippa Addis, Clara Locatelli, Martina Penati, Sara Fusar Poli, Valentina Monistero, Lucia Zingale, Nicola Rota, Claudia Gusmara, Renata Piccinini, Paolo Moroni, Valerio Bronzo

**Affiliations:** 1 Department of Veterinary Medicine and Animal Science - DIVAS, University of Milan, Lodi, Italy; 2 Laboratorio di Malattie Infettive degli Animali - MiLab, University of Milan, Lodi, Italy; 3 Agribovis s.r.l, Meda, Italy; 4 Quality Milk Production Services (QMPS), Cornell University, Ithaca, USA

**Keywords:** Non-*aureus* staphylococci, Coagulase-negative staphylococci, Mammaliicocci, MALDI-TOF MS, Mastitis, Dairy ruminant milk

## Abstract

Non-*aureus* staphylococci and mammaliicocci (NASM) are associated with bovine mastitis and increased milk somatic cell count (SCC) but their relationships with mammary gland health at the species level are not clearly defined. Regional differences have also been reported in their specific prevalence. The implementation of MALDI-TOF MS in milk microbiology is generating large and dependable datasets with the potential of providing useful epidemiological information. We present the retrospective analysis of 17,213 milk samples sent to our laboratory in 2021–2022, including 13,146 quarter samples from cows with subclinical (SCM) or clinical mastitis (CM) from 104 farms, and 4,067 composite herd survey (HS) samples from 21 farms. NASM were isolated from 21.12% of SCM, 11.49% of CM, and 15.59% of HS milk samples. The three most frequently identified NASM in SCM milk were *Staphylococcus chromogenes* (33.33%), *S. haemolyticus* (26.07%), and *S. epidermidis* (10.65%); together with *S. microti* and S. *hyicus*, these species were significantly more prevalent in quarters with SCM (p < 0.05). The three most frequently identified NASM in CM milk were *S. chromogenes* (31.69%), *S. haemolyticus* (21.42%), and *Mammaliicoccus sciuri* (18.38%), although no significant associations were found between these NASM species and CM. The three most frequently identified NASM in HS milk were *S. chromogenes* (44.49%), *S. epidermidis* (17.84%), and *S. haemolyticus* (17.23%), with *S. chromogenes* being isolated in all the farms sending HS milk (100%). In conclusion, this retrospective study provides the first information on the NASM species isolated from cow milk in Italy, expanding our knowledge on the epidemiology of NASM at the species level and providing further insights into their relationships with mammary gland health in modern dairy farms.

**Supplementary Information:**

The online version contains supplementary material available at 10.1007/s11259-023-10187-x.

## Introduction

Non-*aureus* staphylococci (NAS), also referred to as coagulase-negative staphylococci (CNS), are the bacteria most frequently isolated from the milk of dairy cows and are estimated to be the leading cause of intramammary infection (IMI) on modern dairy farms. Although traditionally considered as minor pathogens compared to *Staphylococcus aureus*, some streptococci, and coliforms, NAS are the bacteria most frequently isolated from udder quarters with subclinical mastitis (SCM) (Vanderhaeghen et al. 2014; De Buck et al. 2021). NAS IMIs can contribute significantly to increasing the milk somatic cell count (SCC) of the herd (Schukken et al. 2009). Even though NAS do not seem to be the main cause of clinical mastitis (CM) in herds with significant farm management problems, they can play a role in CM development in well-managed herds (Schukken et al. 2009). The relevance of these pathogens for animal welfare and dairy production quality is evident, but their role and impact on mammary gland health is not completely understood (De Buck et al. 2021), as the species belonging to the genus *Staphylococcus* are also reported as prominent members of the core milk microbiota of dairy animals as well as of mother’s milk (Addis et al. 2016; Oikonomou et al. 2020). In a review of dairy cow mammary microbiota, staphylococci have been mentioned as components of the healthy colostrum, milk, teat canal, and teat apex microbiota, with different potential sources (Derakhshani et al. 2018).

Over 50 species of staphylococci have been described (De Buck et al. 2021) whose taxonomy and pathogenesis are the subjects of an active field of study. Based on 16S rDNA analysis, five *Staphylococcus* spp. have been reclassified into the novel *Mammaliicoccus* genus, namely *S. sciuri*, *S. fleurettii*, *S. lentus*, *S. stepanovicii*, and *S. vitulinus*, with *M. sciuri* as the type species (Madhaiyan et al. 2020), leading to the suggested use of NASM (non-aureus staphylococci and mammaliicocci), as their collective acronym. Different authors have reported on differences among the various species of NASM in terms of host niche adaptation and roles in udder health, as some are more frequently found as commensals while others as environmental contaminants (Hamel et al. 2020). Some NASM may therefore be more associated with the mammary gland, while others may have lower tropism for this organ and be more associated with environmental sources (De Visscher et al. 2014). According to various authors, *S. chromogenes, S. simulans*, *S. xylosus*, *S. haemolyticus*, *S. epidermidis*, and *M. sciuri* are the NASM species most frequently isolated from cow milk (Vanderhaeghen et al. 2014; Condas et al. 2017; De Buck et al. 2021) but their role in udder health ranges from clearly pathogenetic to potentially protective effects against IMI by major mastitis pathogens (Piepers et al. 2010; Derakhshani et al. 2018). Further studies on NASM epidemiology at the species level and on their relationships with udder health are therefore necessary to support more meaningful herd management decisions.

In milk bacteriology, NASM are typically identified only at the genus level and reported to vets and farmers as CNS, as this is economically more sustainable and generally considered sufficient information for routine herd management. However, this has limited our knowledge in terms of circulating species. Most data on the NASM species distribution have been collected through molecular identification methods given the limited reliability of biochemical identification (Condas et al. 2017; De Buck et al. 2021; Rosa et al. 2022). Recently, MALDI-TOF MS is increasingly available in veterinary microbiology, enabling to obtain reliable and affordable species identification (Hulland et al. 2018; Rosa et al. 2019), providing a valuable opportunity for retrospective epidemiological studies with associated information on udder health and management variables. Accordingly, the studies carried out by MALDI-TOF MS in several countries have contributed to defining the local ecology and epidemiology of NASM (De Buck et al. 2021), although for many others including Italy specific information was not available.

With these premises, we carried out a retrospective analysis on the NASM isolated from the milk of udder quarters with SCM and CM and from herd survey (HS) composite milk samples sent to the Animal Infectious Disease Laboratory (MiLab) at the University of Milan, beginning from the date of MALDI-TOF MS implementation in milk bacteriology. The NASM genus and species prevalence and their relationships with mastitis are presented and discussed.

## Materials and methods

### Data analysis

Our sample base was the milk samples sent to the MiLab laboratory since May 2021 (implementation of MALDI-TOF MS for microbial identification) and until November 2022. Data about farm ID, sampling dates, sample types, and microbiological results had been recorded into a Microsoft Access database. Data relating to quarter samples with associated CM or SCM information, and data relating to composite samples obtained at the first herd survey (HS) sampling carried out in the farm (before any management decision) were extracted in Microsoft Excel for the purposes of this study. The extracted data were elaborated using pivot tables and functions embedded in Excel for generating descriptive tables and plots. Statistical analysis was carried out using SPSS 28.0 (IBM, SPSS, Armonk, USA).

The relative prevalence of NASM species in clinical and subclinical mastitis was compared using a multinomial logistic regression model, using as a reference categories the subclinical outcome, estimating the parameters with Wald statistics. p value of < 0.05 and < 0.01 was considered significant for the analyses.

### Milk sample data included in the study

Based on the above specifications, the data relating to 17,213 milk samples were extracted from the laboratory database for the purposes of this work. The dairy farms were located mostly in Northern Italy and consisted mainly of Holstein-Friesian cows housed in free-stall barns. The 13,146 quarter milk samples with associated SCM or CM information had been sent throughout the study period by 104 different farms. Milkers and field technicians had been trained to recognize signs of CM (swelling, redness, pain of udder quarters and alterations of milk such as clots, abnormal colours and consistency) during udder preparation before each milking session. Cows with high SCC at last Dairy Herd Improvement recording were tested through California Mastitis Test (CMT): quarters that resulted only positive to CMT without signs of CM were labelled as SCM samples, while quarters that matched the above criteria for CM were labelled as CM samples. Due to the repetition of sampling for invalid results or follow-up assessments, about 9% of them were replicate collections from one quarter (typically two replicates). The 4,067 composite HS samples had been sent by 21 farms (one sampling per farm). The number of cows sampled per farm ranged from 79 to 497 (mean ± SD = 193.67 ± 98.29). Composite HS udder samples had been collected during total herd samplings for farm survey or contagious pathogen detection. All the milk samples had been collected according to the National Mastitis Council guidelines (Adkins et al. 2017) by trained personnel and sent to the laboratory under cooled conditions. Before sampling, teats were cleaned with pre-dipping disinfectant, and the apex was scrubbed with alcohol-containing disposable wipes. After discarding the first streams of milk, 10 mL was collected aseptically from the quarter into sterile vials. For composite samples, the procedure was applied to all 4 teats, and around 40 mL of milk was collected in the same vial.

### Bacteriological culture

Bacteriological cultures were performed according to the National Mastitis Council guidelines (Middleton et al. 2017). Briefly, milk was spread on blood agar (Microbiol, Cagliari, Italy) and incubated at 37 °C in aerobic conditions. Plates were read after 24 and 48 h and the milk was classified as negative, contaminated, or positive based on the definitions provided by the National Mastitis Council (Adkins et al. 2017). Isolated colonies were identified by MALDI-TOF MS as previously described (Monistero et al. 2021; Rosa et al. 2022). Spectra were processed with the MBT Compass® Library Revision H (2021), covering 3893 species/entries. *Mammaliicoccus* spp. were defined as *Staphylococcus* spp. in the library revision implemented at the time of this study but were reported as *Mammaliicoccus* spp. in this work given their recent reclassification (Madhaiyan et al. 2020). A cutoff log score ≥ 1.7 was considered for species identification as this approach has been validated by various authors as appropriate and highly accurate for bovine NAS (Han et al. 2015; Cameron et al. 2017; Mahmmod et al. 2018; Conesa et al. 2020), and confirmed in our previous study in a comparative assessment with gap PCR-RFLP (Rosa et al. 2022).

## Results

### Microbiology results for quarter and composite milk samples

The milk microbiology results obtained on all 17,213 samples are detailed in Table 1 according to the milk type. Out of 13,146 milk samples collected from single quarters, 64.16% (8,435) were microbiologically positive, 27.90% (3,669) were negative, and 7.94% (1,043) were contaminated. Based on the type of mastitis, 21.12% (1,168) of SCM and 11.49% (2,714) of CM quarters were positive for NASM, respectively. Out of 4,067 composite HS samples from 21 herds, 35.65% (1,450) were positive, 56.16% (2,284) were negative, and 8.19% (333) were contaminated. The composite HS samples positive for NASM were 15.59% (634).

**Table 1:** Milk microbiology results obtained on the 17,213 samples including milk from quarters with subclinical mastitis, milk from quarters with clinical mastitis, and composite herd survey milk

Subclinical quarter milk	Number of isolates (%)	Clinical quarter milk	Number of isolates (%)	Composite herd survey milk	Number of isolates (%)
NASM	1,168 (21.12%)	*S. uberis*	1,415 (18.58%)	NASM	634 (15.59%)
*S. uberis*	610 (11.03%)	*E. coli*	929 (12.20%)	*S. uberis*	280 (6.88%)
*S. aureus*	374 (6.76%)	NASM	875 (11.49%)	*S. aureus*	142 (3.49%)
*Corynebacterium* spp.	288 (5.21%)	*Streptococcus spp.*	420 (5.52%)	*Corynebacterium* spp.	77 (1.89%)
*Streptococcus* spp.	208 (3.76%)	Other Gram-negatives	269 (3.53%)	*Enterococcus* spp.	54 (1.33%)
*Serratia* spp.	197 (3.56%)	*Serratia* spp.	212 (2.78%)	*Streptococcus* spp.	53 (1.30%)
*Enterococcus* spp.	193 (3.49%)	*Enterococcus* spp.	160 (2.10%)	Other Gram-negatives	48 (1.18%)
*E. coli*	149 (2.69%)	*S. aureus*	158 (2.07%)	Bacilli	44 (1.08%)
Other Gram-negatives	142 (2.57%)	*Corynebacterium* spp.	144 (1.89%)	*Serratia* spp.	38 (0.93%)
Fungi	133 (2.40%)	*Klebsiella* spp.	107 (1.41%)	*S. agalactiae*	27 (0.66%)
Bacilli	84 (1.52%)	Bacilli	83 (1.09%)	*E. coli*	24 (0.59%)
*Klebsiella* spp.	27 (0.49%)	Fungi	50 (0.66%)	*Prototheca* spp.	15 (0.37%)
*S. agalactiae*	13 (0.24%)	*Prototheca* spp.	9 (0.66%)	Fungi	8 (0.20%)
*Prototheca* spp.	9 (0.16%)	*S. agalactiae*	8 (0.11%)	*Klebsiella* spp.	6 (0.15%)
Contaminated	347 (6.27%)	Contaminated	696 (9.14%)	Contaminated	333 (8.19%)
Negative	1,589 (28.73%)	Negative	2,080 (27.31%)	Negative	2,284 (56.16%)
Total	5,531 (100.00%)	Total	7,615 (100.00%)	Total	4,067 (100.00%)

### NASM species identified in mastitis and herd survey milk

Species information could be retrieved from the laboratory database for 2,195 NASM isolates. A total of 20 different NASM species were identified and are reported in Table 2 in decreasing order with their respective MALDI-TOF MS identification log score.

**Table 2:** Species identification obtained for the 2,195 identified NASM isolates, listed in decreasing order, with the respective average MALDI-TOF MS Log score

NASM species	N (%)	Average Log score
*S. chromogenes*	776 (35.35%)	2.15
*S. haemolyticus*	496 (22.60%)	1.93
S. sciuri*	262 (11.94%)	1.95
*S. epidermidis*	225 (10.25%)	2.07
*S. xylosus*	209 (9.52%)	1.97
*S. microti*	53 (2.41%)	1.97
*S. arlettae*	43 (1.96%)	1.76
*S. simulans*	40 (1.82%)	2.09
*S. equorum*	35 (1.59%)	2.00
*S. hyicus*	16 (0.73%)	1.93
*S. warneri*	8 (0.36%)	1.99
*S. hominis*	6 (0.27%)	2.05
*S. capitis*	6 (0.27%)	2.03
*S. gallinarum*	6 (0.27%)	1.79
*S. succinus*	4 (0.18%)	1.89
*S. cohnii*	4 (0.18%)	1.85
*S. lentus*	2 (0.09%)	1.84
*S. equinus*	2 (0.09%)	1.8
*S. caprae*	1 (0.05%)	1.84
*S. kloosii*	1 (0.05%)	1.7

*Mammaliicoccus sciuri is referred as *Staphylococcus sciuri* in the Bruker Biotyper® library

Table 3 details the NASM species identified in the different sample types. The 1696 NASM isolated from SCM and CM quarters included 19 different species; 5 of them accounted for almost 90% of all identifications, namely *S. chromogenes* (32.67%), *S. haemolyticus* (24.17%), *M. sciuri* (13.62%), *S. xylosus* (9.96%), and *S. epidermidis* (8.02%). Other less frequently identified species were *S. microti* (2.71%), *S. equorum* (2.30%), *S. arlettae* (2.00%), and *S. simulans* (1.83%). Minor NASM species including *S. hyicus*, *S. capitis*, *S. gallinarum*, *S. equinus*, *S. hominis*, *S. cohnii*, *S. kloosii*, *S. succinus*, and *S. warneri* accounted for the remaining 2.72%. *S. chromogenes* predominated in both SCM and CM quarters, with a slightly higher percentage in SCM vs. CM (33.33% vs. 31.69%). The second most prevalent NASM species was *S. haemolyticus*, also higher in SCM than CM (26.07% vs. 21.42%). Concerning the third most prevalent NASM, *M. sciuri* predominated in CM (18.38% in CM vs. 10.35% in SCM) while *S. epidermidis* predominated in SCM (10.65% in SCM vs. only 4.20% in CM) (Table 2). Based on the results obtained on quarter milk, *S. chromogenes* (p < 0.01), *S. epidermidis* (p < 0.01), *S. haemolyticus* (p < 0.01), *S. microti* (p < 0.01), and S. *hyicus* (p < 0.05) were significantly more prevalent in SCM (Supplementary Table).

**Table 3:** NASM species identified by MALDI-TOF MS in subclinical quarter milk, clinical quarter milk, and composite herd survey milk

Subclinical quarter milk	Number of isolates (%)	Clinical quarter milk	Number of isolates (%)	Composite herd survey milk	Number of isolates (%)
*S. chromogenes*	335 (33.33%)	*S. chromogenes*	219 (31.69%)	*S. chromogenes*	222 (44.49%)
*S. haemolyticus*	262 (26.07%)	*S. haemolyticus*	148 (21.42%)	*S. epidermidis*	89 (17.84%)
*S. epidermidis*	107 (10.65%)	M. sciuri*	127 (18.38%)	*S. haemolyticus*	86 (17.23%)
M. sciuri*	104 (10.35%)	*S. xylosus*	94 (13.60%)	*S. xylosus*	40 (8.02%)
*S. xylosus*	75 (7.46%)	*S. epidermidis*	29 (4.20%)	M. sciuri*	31 (6.21%)
*S. microti*	42 (4.18%)	*S. arlettae*	21 (3.04%)	*S. simulans*	9 (1.80%)
*S. arlettae*	18 (1.79%)	*S. equorum*	19 (2.75%)	*S. microti*	7 (1.40%)
*S. simulans*	18 (1.79%)	*S. simulans*	13 (1.88%)	*S. arlettae*	4 (0.80%)
*S. equorum*	15 (1.49%)	*S. microti*	4 (0.58%)	*S. hominis*	2 (0.40%)
*S. hyicus*	11 (1.09%)	*S. hyicus*	4 (0.58%)	*S. succinus*	2 (0.40%)
*S. capitis*	5 (0.50%)	*S. warneri*	4 (0.58%)	*S. capitis*	1 (0.20%)
*S. hominis*	4 (0.40%)	*S. gallinarum*	3 (0.43%)	*S. caprae*	1 (0.20%)
*S. warneri*	2 (0.20%)	*S. lentus*	2 (0.29%)	*S. cohnii*	1 (0.20%)
*S. gallinarum*	2 (0.20%)	*S. succinus*	2 (0.29%)	*S. equorum*	1 (0.20%)
*S. cohnii*	2 (0.20%)	*S. capitis*	1 (0.14%)	*S. gallinarum*	1 (0.20%)
*S. equinus*	2 (0.20%)	*S. cohnii*	1 (0.14%)	*S. hyicus*	1 (0.20%)
*S. kloosii*	1 (0.10%)	-	-	*S. warneri*	1 (0.20%)
Total	1,005	Total	691	Total	499

*Mammaliicoccus sciuri is referred as *Staphylococcus sciuri* in the Bruker Biotyper® library

The 499 NASM isolated from composite HS samples included 17 different species. *S. chromogenes* accounted for the largest proportion (44.49%). Other 4 species were highly represented including *S. epidermidis* (17.84%), *S. haemolyticus* (17.23%), *S. xylosus* (8.02%), and *M. sciuri* (6.21%). Collectively, these 5 species accounted for 93.79% of all identifications. These were followed by *S. simulans* (1.80%), *S. microti* (1.40%), *S. arlettae* (0.80%), and other 9 NASM species representing the remaining 2%.

### Pathogen distribution and NASM species abundance by farm

**Fig. 1:**
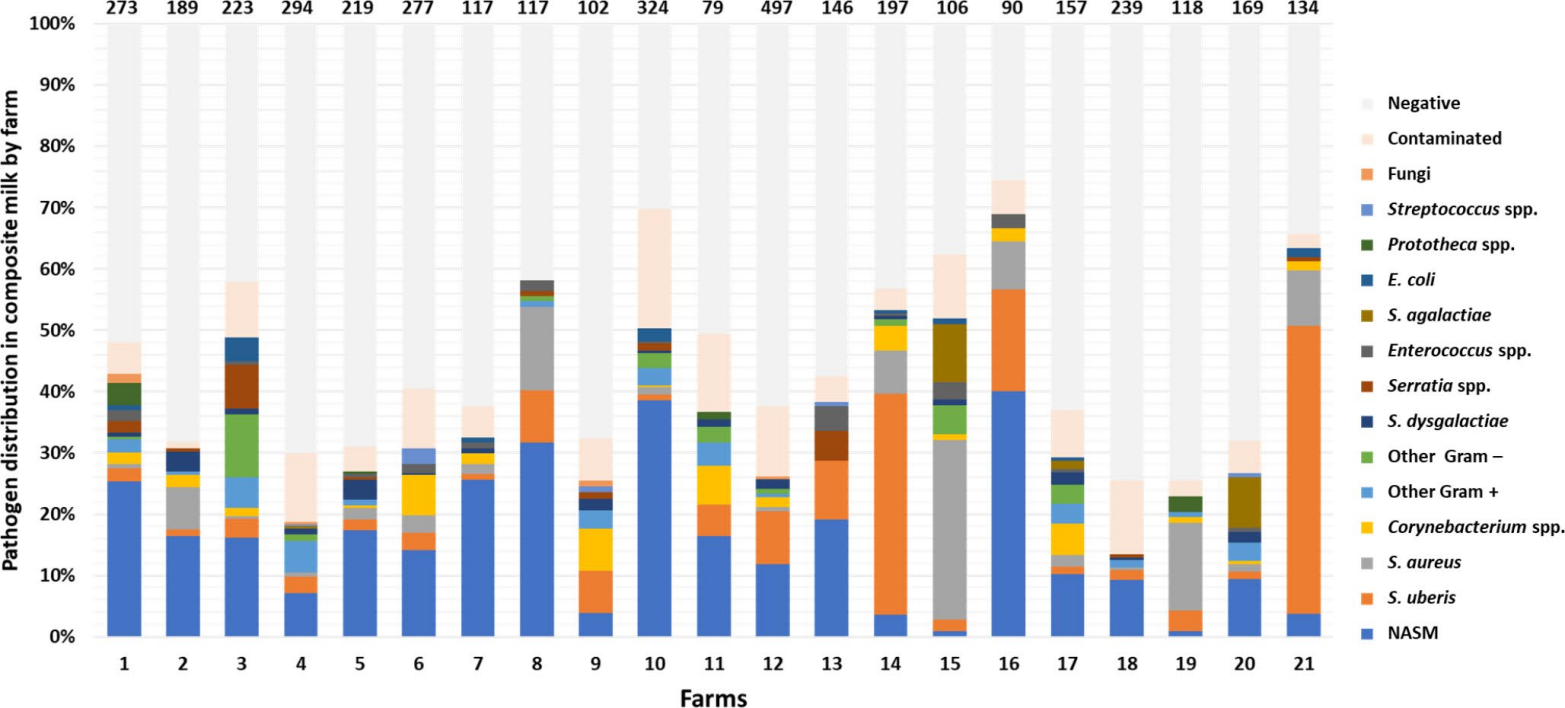
Relative percent distribution of the milk microbiology results obtained on composite herd survey milk according to the contributing farm. The farm number is indicated in the X axis. The total number of samples collected in each farm is indicated above the corresponding histogram bar

The NASM prevalence in each farm sending HS composite samples is illustrated in Fig. 1 together with the main pathogen classes. The relative percent abundance of NASM compared to the other pathogens ranged from 78.95% in farm 7 to 1.82% in farm 15. The farms with the lowest prevalence of NASM had the highest prevalence of major pathogens: farm 15 (1.82% for NASM vs. 56.36% for *S. aureus*), farm 19 (3.70% for NASM vs. 62.96% for *S. aureus*), farm 21 (5.88% for NASM vs. 74.12% for *S. uberis*) and farm 14 (6.67% for NASM vs. 67.62% for *S. uberis*). On the other hand, the farms with the highest NASM prevalence had a very low prevalence of major pathogens.

**Fig. 2:**
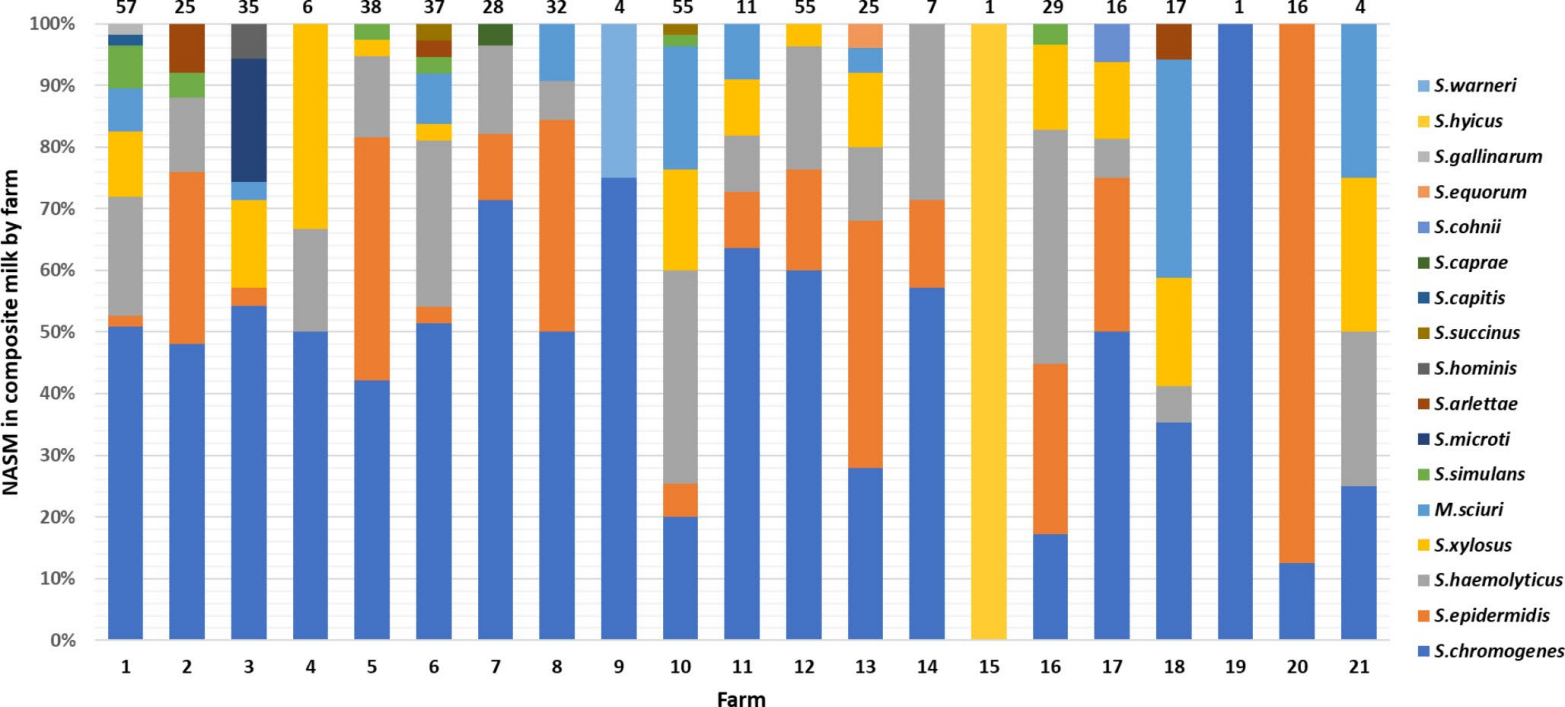
Relative percent distribution of NASM species identified in composite herd survey milk according to the contributing farm. The farm number is indicated below each bar, while the total number of NASM identified on the farm is indicated above the corresponding histogram bar

The distribution of the different NASM species identified on each farm sending HS composite samples is illustrated in Fig. 2. *S. chromogenes* was isolated in all 21 farms (100%). *S. chromogenes* was the main NASM in 14 out of 21 farms (66.66%), representing 100–35.29% of all species. *S. haemolyticus* was isolated in 16 out of 21 (76.19%) farms representing 37.93–5.88% of all species. *S. epidermidis* was isolated in 15 out of 21 (71.43%) farms representing 87.50–1.75% of all species. *S. xylosus* was isolated in 13 out of 21 (61.90%) farms representing 33.33–2.63% of all species. Other relatively frequent NASM species were *M. sciuri*, isolated in 9 out of 21 (42.86%) farms and representing 35.29–2.86% of all species, and *S. simulans*, isolated in 6 out of 21 (28.57%) farms and representing 7.02–1.82% of all species. Finally, *S. arlettae* was isolated in 3 out of 21 (14.29%) farms and represented 8.00–2.70% of all species. All the other NASM were isolated each one only on a single farm (4.76%).

## Discussion

The relevance of NASM for animal welfare and dairy production quality has become increasingly evident in the last few years, and these pathogens are estimated to be the leading cause of IMI on modern dairy farms. Based on our study, NASM were the most prevalent microbial group identified in bovine quarters with SCM, accounting for 21.12%, followed by *Streptococcus uberis* with 11.03%, and by *Corynebacterium* spp. with 5.21%. All other pathogen classes were below 5%. In line with their suggested role in CM, we observed NASM in 11.49% of clinical cases, after the major environmental pathogens *S. uberis* (18.58%, reaching 24.1% when considering all *Streptococcus* spp.) and *E. coli* (12.20%). NASM were also the most frequently identified bacteria in composite milk sent to our laboratory for herd survey purposes (15.59%).

According to a recent systematic review, the most prevalent NASM species isolated from dairy ruminant milk are *S. chromogenes*, *S. xylosus*, and *S. haemolyticus* (Ruiz-Romero and Vargas-Bello-Pérez 2023). A comprehensive review (De Buck et al. 2021) summarized the three most frequently isolated NASM species from quarters with SCM or CM in various countries; when the quarter milk of more than one herd was examined, *S. chromogenes* emerged as the most prevalent species in Belgium, The Netherlands, Finland, Canada, USA, Argentina. In a large-scale Canadian study (Condas et al. 2017), 6.30% of 98,233 quarter-milk samples were associated with NASM IMI; *S. chromogenes*, *S. simulans*, *S. haemolyticus*, *S. xylosus*, and *S. epidermidis* were the most common among 23 different identified species. These data were in line with our findings, being *S. chromogenes* the most prevalent NASM in Italian dairy farms followed by *S. haemolyticus*, *M. sciuri*, *S. xylosus*, and *S. epidermidis*. However, in addition to farm management and regional variables, it should be considered that in the previous epidemiological studies NASM identification was obtained mainly with molecular methods such as partial sequencing of the *rpoB* housekeeping gene, and not by MALDI-TOF MS.

In a German study carried out on quarter milk samples employing MALDI-TOF MS for species identification (Hamel et al. 2020), S. *chromogenes* and *S. simulans* were associated with IMI in over 90% of cases, while *S. warneri*, *S. xylosus*, *S. microti*, *S. haemolyticus*, and *S. succinus* were suggested to be frequent causes of IMI as well as contaminants. Their findings concerning *S. chromogenes* were in line with our study. Specifically, we found *S. chromogenes* as the most frequently identified NASM in all sample types (SCM and CM quarter milk and composite milk), accounting for almost half of all identifications (44.49%) in composite milk. *S. haemolyticus* was significantly associated with the presence of SCM and it was the second identified species in quarter milk, while *S. epidermidis* was the second identified in composite milk. The third most prevalent species changed according to the sample type, with *S. epidermidis* in SCM, *M. sciuri* in CM, and *S. haemolyticus* in composite milk.

Our sample set included HS composite milk from 21 herds, enabling us to investigate the presence of NASM, their relationships with other pathogens, and the NASM species distribution at the farm level. NASM were present in all herds at very different levels; the farms with the lowest prevalence of NASM had the highest prevalence of major pathogens, while the farms with the highest NASM prevalence had the lowest prevalence of major pathogens. A high prevalence of *S. uberis* and *S. aureus* was especially associated with a low NASM prevalence within the herd. This is in line with the reported potentially protective effect of NASM against major pathogens, as well as with their emergence as IMI agents when infections by major pathogens are under control (Piepers et al. 2010, 2013; Ruegg 2017).

Being a retrospective survey based on laboratory data, it is difficult to investigate the impact of farm characteristics on the NASM species prevalence and distribution. As these variables are known to play an important role in within-farm epidemiology (Piessens et al. 2011; De Visscher et al. 2014; De Buck et al. 2021), future studies will be required to establish their correlations with NASM diffusion, considering a greater number of samples and farms to better understand the prevalence of minor NASM species. Strain variables can also play relevant roles in within-farm epidemiology, but these cannot be resolved with the currently available MALDI-TOF MS technology and instrumentation.

Expanding on the limitations of MALDI-TOF MS as the microbial identification method, the average identification log score was lower than 2.00 for several NASM species. A cutoff log score ≥ 1.7 has been indicated by various authors as appropriate and highly accurate for NAS species identification (Han et al. 2015; Cameron et al. 2017; Mahmmod et al. 2018; Conesa et al. 2020), and it has been confirmed in our previous study in a comparative assessment with gap PCR-RFLP (Rosa et al. 2022), but increasing this value would be advisable to increase the reliability of epidemiological data. One of the species showing an average Log score < 2.00 was *S. haemolyticus*, one of the most prevalent NASM, as recently observed also for sheep and goat milk isolates (Rosa et al. 2022) S. *haemolyticus* has a high genomic variability as well as a high similarity to the 16 S rRNA gene with other species, which might be reflected in a phenotypic similarity of ribosomal proteins and therefore of MALDI-TOF mass spectra. A Polish study (Wanecka et al. 2019) reported that the 16 S rRNA gene of 27 out of 33 *S. haemolyticus* isolates shared 99.5–100% similarity with other species including *S. epidermidis*. Some *S. haemolyticus* were recently reclassified as *S. borealis* (Pain et al. 2020), and spectra for this latter species were not present in the library release implemented during our study. The library updates released by the instrument manufacturers may help solve this issue but improving in-house spectrum libraries with bovine isolates is also suggested for increasing the reliability of species identification. The high similarity of *S. microti* with other species such as *S. rostri* may also require further investigations (Rosa et al. 2022). Other prevalent species that may benefit from a spectrum library expansion are *S. sciuri* and *S. xylosus*.

In conclusion, this study expanded our knowledge of the relationships of NASM species with the presence of mastitis and provided the first information on NASM epidemiology in Italian dairy herds. A better understanding of their roles in mammary gland health at the species level will hopefully lead to making more meaningful management decisions when NASM are isolated from the milk of cows with mastitis.

### Electronic supplementary material

Below is the link to the electronic supplementary material.

Supplementary Material 1

## Data Availability

Not applicable.
