# Supplementary material for: Non-aureus staphylococci and mammaliicocci isolated from bovine milk in Italian dairy farms: a retrospective investigation
Source: Vet Res Commun. 2023 Aug 10;48(1):547–54. doi: 10.1007/s11259-023-10187-x (PMC10811067; doi:10.1007/s11259-023-10187-x)
Supplement: Supplementary file 1 — Supplementary Material 1 [file 11259_2023_10187_MOESM1_ESM.pdf]

Non-*aureus* staphylococci and mammaliicocci isolated from bovine milk in Italian dairy farms: a retrospective investigation.

Maria Filippa Addis[1,2*], Clara Locatelli[1], Martina Penati[1], Sara Fusar Poli[1], Valentina Monistero[1], Lucia Zingale[1], Nicola Rota[3], Claudia Gusmara[1], Renata Piccinini[1], Paolo Moroni[1,2,4], Valerio Bronzo[1,2]
[1]Department of Veterinary Medicine and Animal Science - DIVAS, University of Milan, Lodi, Italy
[2]Laboratorio di Malattie Infettive degli Animali - MiLab, University of Milan, Lodi, Italy
[3]Agribovis s.r.l., Meda, Italy
[4]Quality Milk Production Services (QMPS), Cornell University, Ithaca, USA

Supplementary Table Results of the application of a multinomial logistic regression model, using as a reference category the subclinical outcome, estimating the parameters with Wald statistics, for assessing the relationships with the NASM species.

Mast_cod[a]		b	Std. Err.	Wald	Ggl	*P* value	Exp(b)	95% conf. interval for Exp(b)	
								Lower limit	Upper limit
CM	Intercept	0.226	0.155	2.127	1	0.145			
	[NASM = *S. arlettae*]	-0.072	0.357	0.040	1	0.841	0.931	0.463	1.872
	[NASM = *S. capitis*]	-1.835	1.106	2.752	1	0.097	0.160	0.018	1.395
	[NASM = *S. chromogenes*]	-0.651	0.178	13.439	1	**0.000**	0.522	0.368	0.739
	[NASM = *S. cohnii*]	-0.919	1.234	0.554	1	0.457	0.399	0.035	4.484
	[NASM = *S. epidermidis*]	-1.531	0.260	34.587	1	**0.000**	0.216	0.130	0.360
	[NASM = *S. equorum*]	0.011	0.379	0.001	1	0.978	1.011	0.481	2.122
	[NASM = *S. gallinarum*]	0.180	0.926	0.038	1	0.846	1.197	0.195	7.348
	[NASM = *S. haemolyticus*]	-0.797	0.186	18.385	1	**0.000**	0.451	0.313	0.649
	[NASM = *S. hyicus*]	-1.237	0.604	4.196	1	**0.041**	0.290	0.089	0.948
	[NASM = *S. microti*]	-2.577	0.756	11.620	1	**0.001**	0.076	0.017	0.334
	[NASM = *M. sciuri*]	-0.026	0.204	0.016	1	0.898	0.974	0.654	1.452
	[NASM = *S. simulans*]	-0.551	0.396	1.942	1	0.163	0.576	0.265	1.251
	[NASM = *S. warneri*]	0.467	0.880	0.282	1	0.595	1.596	0.285	8.950
	[NASM = *S. xylosus*]	0.072	0.357	0.04	1	0.841	1.074	0.534	2.161
